# Traditional and Sustainable Methods for the Synthesis of Quinoline Derivatives as Anticancer Agents (2019–Present): A Comprehensive Review

**DOI:** 10.2174/0118715206411442251115061336

**Published:** 2026-01-12

**Authors:** Gajendra S. Thakur, Ajay K. Gupta, Yogesh Vaishnav, Sivakumar Annadurai, Sanmati K. Jain

**Affiliations:** 1 Department of Pharmacy, Drug Discovery and Research Laboratory, Guru Ghasidas Vishwavidyalaya (A Central University), Bilaspur, 495009, Chhattisgarh, India;; 2 Department of Pharmacognosy, College of Pharmacy, King Khalid University, Alfaraa, 61421, Saudi Arabia

**Keywords:** Anticancer quinolines, green synthesis, hybrid quinoline derivatives, Sustainable chemistry, One-pot multicomponent reactions, Heterocyclic compounds

## Abstract

**Introduction:**

Heterocyclic compounds are widely utilized in the development of anticancer medications due to their diverse structures and ability to interact with multiple biological targets within cancer cells. Quinoline is a heterocyclic compound and an essential compound in the domains of industrial and pharmaceutical chemistry because of its various pharmacological effects. Researchers are developing new traditional, synthetic, and innovative green approaches to synthesize mono- or poly-substituted quinoline derivatives for anticancer activity.

**Methods:**

A comprehensive literature survey was conducted using multiple databases, including Google Scholar, PubMed, SpringerLink, ScienceDirect, and others, to investigate the existing literature on synthetic strategies for various quinoline derivatives. This review article intends to present a summary of various traditional synthetic methods alongside innovative green approaches.

**Results:**

Many researchers have demonstrated that quinoline derivatives can be synthesized using various methods, including traditional techniques, hybrid approaches with heterocyclic structures, and innovative green synthetic methods, as well as elucidating their structure-activity relationships for potential use as anticancer agents. The majority of traditional synthetic methods rely on hazardous chemicals, low reaction rates, high temperatures, and high pressures. Currently, the green chemistry approach produces eco-friendly, economical, high-yield, pure, and outstanding products in the fields of industry and pharmaceuticals.

**Discussion:**

This section explores various affordable and eco-friendly synthetic techniques that produce potent and specific quinoline compounds, intended for use as anticancer agents.

**Conclusion:**

The progress demonstrated in the green synthetic methods and the development of quinoline-based compounds as new treatment options could aid in identifying new and effective quinoline derivatives for cancer treatment in the future.

## 1 INTRODUCTION

Considering the cancer's growing global impact, fighting it has become an important health issue in the twenty-first century. According to the International Agency for Research on Cancer (IARC), there are going to be around 9.7 million cancer-related deaths and 20 million new cases of cancer diagnosed globally in 2022 [1-3]. Malignant disease refers to the spread of cancer cells. Various treatment options are available for management, ranging from chemotherapy to radiation therapy, including surgery and hormonal therapy. However, chemotherapy remains the most widely prescribed therapeutic option for cancer management. Chemotherapy is beneficial to a limit, but most anticancer drugs have severe dose-related side effects as well as damage to normal cells and tissue, resulting in systemic toxicity. An anti-cancer medication faces several challenges, including poor solubility, low permeability, reduced biocompatibility, increased side effects, and toxicity. Worldwide, cancer is the second most prevalent cause of death [4, 5].

Quinoline, a nitrogen-containing heterocyclic aromatic molecule with a weak tertiary base, is a key component of natural alkaloids and a significant class of heterocyclic substances that may form salts with acids and remain in electrophilic substitution processes. As the name signifies, benzopyridine is produced when a benzene ring is fused to the α and β locations of a pyridine ring. The compounds containing the quinoline moiety, such as cabozantinib, neratinib, lenvatinib, foretinib, irinotecan, and topotecan, are the marketed anticancer agents shown in Fig. (**1**). The quinoline scaffold-containing anticancer drug has been extensively and effectively explored. In this discussion, we are also addressing candidates involved in clinical trials and their implications for the near future, which significantly contribute to advancements in drug discovery [6-8]. In 2020, Mohamed and Abuo-Rahma also reported several molecules containing the quinoline framework as anticancer agents, such as GSK-1059615, PF-04217903, Silmitasertib-CX-4945, and AMG-319, which are compounds under clinical trials [9, 10]. The quinoline-containing anticancer agents now undergoing clinical trials are shown in Fig. (**2**).

Various traditional methods have been employed in the synthesis of the quinoline framework. The Skraup synthesis is the most widely recognized approach, which involves heating aniline or its derivatives with glycerol in the presence of nitrobenzene, sulfuric acid, and ferrous sulfate. Both large and small scales extensively utilized this technique for quinoline synthesis [11-15]. Additionally, other methods, such as the Doebner-von Miller [16-19], Conrad-Limpach-Knorr [20], and Combes methods [21, 22], exist. Alternative synthesis routes for quinoline derivatives include Friedländer, Pfitzinger, and Niementowski synthesis, which utilize ortho-substituted derivatives. Nonetheless, it is essential to incorporate and implement innovative green chemistry techniques into our conventional processes to enhance yield, selectivity, productivity, energy efficiency, lifespan, and time management [23-30].

The modern definition of “green chemistry,” also known as “sustainable technology,” refers to a chemical process that utilizes raw resources, minimizes waste, and avoids the use of hazardous and toxic reagents and solvents. Additionally, a low number of solvents, reagents, and promoters should be used to obtain excellent product selectivity at a cost-effective reaction [31-33]. Paul Anastas and John Warner formulated twelve principles of “green chemistry,” which include designing for energy efficiency, employing safe catalysts, ensuring atom economy, utilizing less hazardous substances, facilitating design for degradation, minimizing derivatives, preventing waste, conducting real-time analysis for pollution control, preventing accidents, adopting sustainable synthetic methods, and utilizing safer solvents and renewable feedstocks [34].

Consequently, this review will encompass recent advancements (primarily from 2019 to the present) in traditional and green synthetic methods, including microwave-assisted synthesis, ultrasound-enhanced synthesis, and synthesis utilizing green catalysts or solvent-free techniques. It will also examine the hybridization of quinoline scaffolds with heterocyclic compounds, such as imidazole, furan, pyrazole, indole, thiazole, and natural products, as anticancer agents, while briefly discussing the general chemistry of quinoline scaffolds, including structure-activity relationships (SAR) related to their anticancer effectiveness [35-40].

## 2 METHODOLOGY

To investigate both traditional and green synthetic methods for quinoline and its derivatives, we reviewed various literature sources from databases and search engines, including Google Scholar, Web of Science, PubMed, SpringerLink, ScienceDirect, ACS Publications, Wiley Online Library, and Taylor & Francis Online. The literature search spanned from 2019 to 2025, employing keywords such as “Quinolines as anticancer agents”, “Quinoline derivative”, “Name reaction for quinolines synthesis”, “Green synthetic approach of quinolines”, and “Conventional methods for quinolines synthesis”, among others. Furthermore, the authors specifically searched for name reactions, including the Skraup synthesis, Doebner-Miller reaction, Doebner synthesis, Riehm synthesis, Gould-Jacobs synthesis, Conrad-Limpach synthesis, Combes’s synthesis, Pfitzinger reaction, Friedlander synthesis, Povarov reaction, Knorr synthesis, Camps synthesis, and Niementowski synthesis of quinolines. We also concentrated on documented pharmacological activities, particularly the *in-vivo* and *in-vitro* effects associated with the anticancer activity of quinolines. A comprehensive review was conducted to identify the most pertinent research and review articles, gathering crucial information to understand the research outcomes related to synthetic methods and the anticancer properties of quinoline derivatives. Over 800 articles were collected, with some being excluded due to a lack of relevance, insufficient availability of materials according to our criteria, low journal impact factors, and the absence of DOI numbers. Ultimately, 147 articles were selected for further analysis.

## 3 CHEMISTRY OF QUINOLINE SCAFFOLD

The chemical formula of quinoline is C_9_H_7_N, classifying it as a heterocyclic aromatic organic compound. It consists of a benzene ring that is fused to a pyridine ring at the α, β position. Isoquinoline, a fused heteroarene, is derived from benzopyridine, where the nitrogen atom is not directly attached to the benzene ring [41-43]. The COVID-19 pandemic has highlighted the necessity for the scientific community to intensify efforts in drug development. Quinoline derivatives, such as 8-aminoquinoline, have garnered significant interest due to the potential role of metabolic transformation in their *in-vivo* activation [44-47]. Table **1** outlines the chemical and physical properties of quinoline [48-50].

### 3.1 General SAR for Quinoline Scaffold as Anticancer Activity

The quinoline ring represents a pharmacophore with considerable potential for drug discovery. Additionally, the SAR indicates that varying substituents at different positions on the quinoline can improve the pharmacological effectiveness of lead compounds containing quinoline. This class of chemicals, quinoline, has been extensively studied due to its diverse biological activities and methods of synthesis. The incorporation of nitrogen atoms significantly enhances the basic properties of quinoline-containing compounds. Moreover, the nitrogen in quinoline can form hydrogen bonds with target enzymes. Another crucial feature that can be leveraged in drug design is the enhancement of water solubility while reducing lipophilicity. The general structure-activity relationship of the quinoline scaffold is illustrated in Fig. (**3**), while the SAR of quinoline derivatives as anticancer agents is detailed in Table **2** [51-54].

## 4 NAME REACTION FOR SYNTHESIS OF QUINOLINE SCAFFOLD

Numerous established reactions, including the Gould-Jacobs, Meth-Cohn, Povarov, Friedlander, Pfitzinger, Conrad-Limpach, Combes, Skraup, and Doebner-von Miller syntheses, often serve as the basis for the most traditional and effective methods for synthesizing quinolines and their derivatives. Quinolines have been synthesized through a variety of conventional techniques or named reactions, all of which rely on the cyclocondensation or cyclization of aryl amines with carbonyl compounds, such as aldehydes and ketones. Table **3** illustrates the conventional process for quinoline production along with its schematic representation [60-82].

## 5 CONVENTIONAL REACTION FOR SYNTHESIS OF QUINOLINE DERIVATIVES AS AN ANTICANCER AGENT

This review article describes the anticancer properties of quinoline derivatives synthesized through various traditional methods. While some of these methods demonstrate effectiveness, they also generate significant waste, necessitate extended reaction durations, involve toxic solvents, require the separation of catalysts from the reaction system, demand uniform catalysts for reuse, and often utilize additional acids along with costlier reagents and oxidants. Moreover, specific processes yield excessive amounts of undesirable byproducts, which must be meticulously minimized or eradicated [83].

Katariya and his team synthesized novel hydrazone-derived quinoline compounds **(1A-F)**. The antitumor effectiveness of these synthesized substances was evaluated against the NCI 60 human cancer cell lines [84]. Iqbal *et al*. report the quinoline-4-carboxylic acid derivatives (**2A-C**) as potent anticancer agents against breast cancer cells (MCF-7), bone marrow cancer cells (K-562), and cervical cancer cells (HeLa), utilizing carboplatin as a reference drug [85]. The design and development of a new thiophene quinoline compound **(3)** bearing 2-chloro substitution were evaluated and reported for anticancer activity against four different human cancer cell lines, such as breast (MCF-7), human cervical cancer (HeLa), and colon cancer (HCT-116), by Othman *et al.* [86].

Quinoline derivatives functionalized with 1,2,4-oxadiazole **(4)** were evaluated against four human cancer cell lines, namely DU-145 (prostate), A549 (lung), and MCF-7 (breast), utilizing etoposide as the positive control, as reported by Kala *et al* [87]. Nasr and his research team synthesized quinoline derivative **(5)** incorporating various heterocycles and chalcone moieties. This compound exhibited significant Topo-1 inhibitory activity, with an IC_50_ value comparable to that of camptothecin, a standard reference drug [88]. Mrozek-Walczykiewicz and others developed compounds of styryl quinoline derivatives (**6A-D**) that showed anticancer effects on colon cancer cells (HCT116) [89].

Özcan and colleagues conducted a study utilizing HeLa, C6, and HT29 cancer cell lines to evaluate the anticancer efficacy of quinoline analogues featuring diverse functional groups (**7A–B**) through various cancer-related assays, employing 5-fluorouracil as a standard drug [90]. Jin *et al*. synthesized quinoline derivatives (**8A-D**) and assessed their efficacy against HeLa, SGC-7901, and L02 cell lines, utilizing 5-FU and methotrexate as reference medications. This study showcased a dual assessment of biological activity for prospective antitumor and antibacterial agents [91].

Fathy and colleagues synthesized a quinoline compound through a one-pot, multicomponent reaction. They generated several tetrahydroquinoline derivatives containing pyrazole and hydrazide groups by reacting 3-amino-tetrahydro-1H-pyrazolo [3,4-b] quinoline with aldehydes and derivatives of indoline-2,3-dione (**9A-C**). These compounds were assessed for their anticancer efficacy against HepG-2 liver cancer cell lines, using doxorubicin as a reference drug [92]. Puskullu and his research team synthesized newer quinoline-hydrazone derivatives (**10**); the *in vitro* cytotoxic effects were assessed in MCF-7 and A549 cell lines using the MTT assay [93].

Köprülü *et al*. presented a novel quinoline and tetrahydroquinoline derivative (**11A-B**) that acts as an inhibitor of the topoisomerase-I enzyme, serving as an anticancer agent [94]. Ren *et al*. synthesized quinoline and acridine derivatives (**12A-B**) that act as tubulin inhibitors, aiming to explore their potential as antitumor agents against various cancer cell lines, such as HeLa, B16-F10, MCF-7, and HepG-2 [95].

Ibrahim *et al*. developed, synthesized, and showcased novel quinoline-based analogues (**13A-E**) of combretastatin A-4, which act as inhibitors of tubulin polymerization. Their mechanistic studies suggest that these analogues may hinder cell migration and tubulin polymerization, leading to G2/M phase arrest. Furthermore, these analogues induce the production of reactive oxygen species in MCF-7 cells and initiate apoptosis *via* the amino-chondral-dependent apoptosis pathway. These results provide a foundation for the continued rational development of potent tubulin polymerization inhibitors for cancer treatment [96].

Zaraei *et al*. developed, synthesized, and evaluated the biological activity of a novel series of diary urea and diarylamine derivatives, featuring a quinoline scaffold (**14A-B**) that incorporates dimethylamino or morpholino side chains, serving as selective C-RAF kinase inhibitors for anticancer applications [97].

Raczkowski *et al*. reported a newer tetrahydroquinoline derivative (**15**) as an antitumor agent [98]. Anwar and colleagues synthesized innovative derivatives of pyrimido-pyridoquinazoline, pyrazoloquinolines, and thiazoloquinoline **(16)** using traditional heating methods, while also employing microwave irradiation to enhance the yield and efficiency of the reactions. These compounds were subsequently tested for their anticancer properties against the HCT-116, HePG-2, and MCF-7 cell lines, with 5-Fluorouracil serving as the positive control [99]. El-Sheref *et al*. developed and synthesized a new series of 4-(1,2,3-triazol-1-yl) quinolin-2(1H)-ones (**17A-D**) through a 1,3-dipolar cycloaddition approach, achieving high yields with high purity. It’s *in-vitro* antiproliferative effect was evaluated in A-549, MCF-7 and HT-29 cells by a caspase-3 assay using erlotinib as a standard drug [100].

Zbancioc *et al*. presented novel benzo[c]quinoline derivatives (**18A-C**) synthesized using traditional heating methods, as well as through microwave irradiation (MWI) and ultrasound (US) irradiation, which enhanced the yield and efficiency of the reactions, showing considerable lethality against the SR leukaemia cell line [101]. Ismail and colleagues presented heterocyclic compounds N-(1-(4-((7-chloroquinolin-4-yl)amino)phenyl)ethylidene)-2-cyanoacetohydrazide (**19A-B**) and evaluated their antiproliferative effects against various cancer cell lines, including PC-3, MCF-7, HCT-116, and HepG2, using doxorubicin as the reference drug [102]. Abu-Hashem *et al.*, synthesized heterocyclic compounds, specifically substituted 6-quinoline-pyridotriazolopyrimidinones (**20**), and evaluated them against the MCF-7 breast carcinoma cell line to investigate their anticancer and antioxidant properties [103].

Hany *et al*. investigated novel 2-(quinoline-4-carbonyl) hydrazide derivatives (**21**) and assessed their cytotoxicity against a breast cancer cell line in comparison to the established medication lapatinib [104]. Zhao *et al*. indicated that the quinoline-2-thione derivative (**22**) KA3D demonstrated efficacy in treating ovarian cancer. They employed the MTT assay for testing and utilized flow cytometry to assess its impact on apoptosis and the cell cycle [105].

Lu *et al*. synthesized and evaluated a range of quinoline-derived dihydrazone derivatives (**23A-C**) for their efficacy against the MCF-7 breast cancer cell line. The results from the MTT assay demonstrated that all compounds exhibited a wide spectrum of antineoplastic activity [106]. The structures of quinoline and its derivatives **(1-23)**, which exhibit anticancer properties, are illustrated in Fig. (**4**-**6**).

## 6 QUINOLINE HYBRID MOLECULES AS ANTICANCER AGENTS

In current drug development, hybridization serves as a suitable strategy that integrates two or more established pharmacophores into a unified scaffold, proving to be an effective method for generating innovative leads with advantageous characteristics [107]. Molecular hybridization presents numerous applications, as it can yield compounds with diverse and/or distinctive mechanisms of action while minimizing adverse effects. The examples of quinolines hybridized with heterocyclic compounds presented in this article aim to provide researchers in the field with an overview of the concept and its potential applications, rather than compiling an exhaustive list of anticancer hybrids [108].

Aly and colleagues synthesized quinoline-pyrazole hybrids (**24**) and assessed the anti-apoptotic properties of these novel compounds in mitigating I/R-induced tissue damage in the colons of rats, utilizing N-acetylcysteine (NAC), a widely recognized anti-apoptotic agent [109]. Venkata and his team synthesized and evaluated a range of 8-bromo-1*H*-1,2,3-triazol-4-yl-2-methylquinoline derivatives (**25A-C**) against human breast cancer (MDA-MB-231) and melanoma cell lines (B16F10) [110].

Othman and colleagues synthesized several novel thiophene-substituted quinoline derivatives (**26A-D**). Additionally, the MTT assay was employed to evaluate the anticancer effectiveness of these compounds against four human cancer cell lines, using 5-FU as the reference drug [111]. Awolade synthesized quinoline-isatin hybrids (**27**) using a click chemistry-based molecular hybridization approach and evaluated their anti-tumor efficacy through MTT assay [112]. Shah *et al*. synthesized specific quinoline-1,3-oxazole hybrid derivatives (**28A-D**) that demonstrated anticancer properties [113].

Kadela-Tomanek and colleagues presented derivatives of quinoline with a 1,4-quinone hybrid (**29A-D**) and assessed their *in vitro* cytotoxic effects on various human cancer cell lines, utilizing doxorubicin as a reference drug [114]. Krstulović *et al*. conducted a study on the synthesis of quinoline-benzimidazole hybrids utilizing two distinct types of triazole methyl-phenoxy linkers (**30**) and evaluated the SAR in HuT78 lymphoma cells [115]. Lavunuri *et al.,* developed quinoline-fused 1,2,3-triazole compounds (**31**) using a reaction that involves copper(I) to combine azides and alkynes, and they tested the effectiveness of these compounds against breast cancer by comparing them to the standard drug 5-FU [116]. El Helw *et al*. designed, synthesized, and assessed a new series of benzo[f]quinoline-derived heterocycles (**32A-B**) as anticancer agents targeting the HCT116 and MCF-7 cancer cell lines [117]. Priya *et al.,* synthesized a novel quinoline derivative featuring substituted piperazine moieties (**33A-G**). The resulting compounds were assessed for their *in vitro* cytotoxic effects against breast cancer (MCF-7), with gefitinib serving as a reference medication [118].

Srinivasa *et al.,* conducted a study on benzimidazole-quinoline hybrid compounds (**34A-B**) and evaluated their cytotoxic effects on melanoma and breast cancer cell lines in comparison to the standard drug, cisplatin [119]. Utilizing multistep synthetic methods, Panduranga and colleagues synthesized several innovative quinoline and sulfonamide compounds, evaluating their anticancer efficacy. Each of these compounds exhibited notable activity against at least one cancer cell line, with compound (**35A-C**) showing the highest level of effectiveness [120]. Gabriele and associates introduced new 4-Piperazinylquinoline hybrids (**36A-D**) and assessed their antiproliferative properties [121]. Taheri and associates presented a new coumarin-quinoline compound **(37)** that showed remarkable yields, mild reaction conditions, and easy availability, demonstrating anticancer activity against ovarian cancer (A2780) cell lines. Doxorubicin served as the standard drug [122].

Yong-Feng *et al*. synthesized quinoline-chalcone derivatives (**38)** and assessed their anti-cancer activity against colon cancer (HCT-116), breast cancer (MCF-7), and gastric cancer (MGC-803) cell lines. This led to the discovery of new compounds exhibiting potential antiproliferative effects on all three cell lines, with 5-FU used as the reference drug [123]. The quinoline/chalcone/1,2,4-triazole hybrids **(39)** developed by Mohassab *et al*. have been evaluated against various cancer cell lines, including colon cancer (HT-29), lung cancer (A549), breast cancer (MCF-7), and pancreatic cancer (PANC-1). The anticancer properties of these quinoline/chalcone compounds, which incorporate the triazole ring, significantly impacted all four cell lines. Doxorubicin served as the reference drug [124].

The antitumor efficacy of fused naphthofuro [3,2-c]quinoline-6,7,12-triones and pyrano [3,2-c] quinoline-6,7,8,13-tetraones **(40)** was assessed against leukemia (CGRF-CEM), lung cancer (A549), and colon cancer (HTC-116) cell lines as reported by Aly *et al* [125]. The structures of quinoline and its derivatives **(24-40)**, which exhibit anticancer properties, are illustrated in Fig. (**7** and **8**).

## 7 SYNTHESIS OF QUINOLINE DERIVATIVES AS AN ANTICANCER AGENT USING GREEN CHEMISTRY APPROACHES

Green chemistry, which promotes sustainability and reduces environmental impact, has revolutionized the synthesis of pharmaceuticals. This review discusses recent advancements in the synthesis of quinoline derivatives for cancer treatment (2019–present) that utilize green chemistry methods, including microwave irradiation, ultrasound, one-pot synthesis, solvent-free processes, green solvents, and catalysts. A novel green chemical approach, as depicted in Fig. (**9**), should be adapted and incorporated into standard practices to enhance yield, selectivity, productivity, energy efficiency, lifespan, and time management. The benefits and some drawbacks of green chemistry techniques are outlined in Table **4** [126-129].

Mokhtar and his team utilized chitosan-coated copper nanoparticles (CS/Cu-NPs) as a catalyst, employing ethanol as a solvent under ultrasonic irradiation to synthesize a range of quinoline derivatives from dimedone **(41)**, substituted aldehydes (**42**), methylene compounds (**43**), and ammonium acetate **(44)**. The compounds 4,6,7,8-tetrahydroquinolin-5(1H)-ones (**45A-D**) demonstrated the highest cytotoxicity, with IC_50_ values ranging from 0.002 to 0.004 mM, compared to staurosporine, a reference drug. This reaction is illustrated in Fig. (**10**) [136].

Mu and colleagues developed various quinoline derivatives by employing 2,6-dichloroquinoline-3-carbaldehyde **(46)** ethyl 2-mercaptoacetate **(47),** and gamma-valerolactone (GVL) in an environmentally friendly solvent within a basic medium. The resulting compounds exhibited notable cytotoxic properties, with compound **(48A-D)** presenting an IC_50_ value of 8.04μM in comparison to the reference drug NSC-87877. This reaction is depicted in Fig. (**11**) [137].

Patel and colleagues synthesized a range of quinoline derivatives using dimedone (**49**), 2,6-dimethoxypyrimidin-4-amine (**50**), and various substituted aldehydes (**51**), employing acetic acid/glacial acetic acid as a catalyst under microwave assistance. The resulting compounds, specifically 5,8,9- and 5,8,9,10-tetrahydropyrimido [4,5-b]quinolin-6(7H)-one (**52A-D**), demonstrated the highest cytotoxic efficacy, with yields ranging from 81% to 86%. This reaction is illustrated in Fig. (**12**) [138]. Alanazi and colleagues synthesized a range of quinoline derivatives from 2,6-di((E)-benzylidene)cyclohexan-1-one (**53**) and 2-cyanoethanethioamide (**54**) using NaOH as a base catalyst and ethanol as a solvent under ultrasonic irradiation. The resulting tetrahydroquinoline derivatives **(55, 56)** demonstrated superior cytotoxic activity when compared to doxorubicin, which served as a standard drug. This reaction is illustrated in Fig. (**13**) [139].

Ramya and colleagues synthesized a quinoline-triazole hybrid from 8-(prop-2-yn-1-yloxy)quinoline (**57**) and 3-azido-N-(3-oxo-1,3-diphenylpropyl)propenamide (**58**), utilizing copper sulfate and tBuOH as catalysts, with DMSO/H_2_O serving as the solvent, through a multicomponent coupling approach and click chemistry. The resulting compound, N-(1-phenylpropyl)-3-(4-((quinolin-8-yloxy)methyl)-1H-1,2,3-triazol-1-yl)propenamide **(59)**, demonstrated significant cytotoxic effects against the human breast cancer cell line MCF-7, exhibiting an IC_50_ value of 8 μM [140]. This reaction is illustrated in Fig. (**14**). A study conducted by Aly *et al.,* detailed the synthesis of various quinoline derivatives from 4-hydroxyquinolin-2(1*H*)-one (**60**) and ethyl (Z)-3-(furan-2-yl)-2-isocyanoacrylate **(61)** utilizing potassium carbonate as a catalyst and ethanol as a solvent under microwave assistance. The resulting compounds, pyrano [3,2-c] quinoline-3-carboxylate derivatives (**62A-H**), demonstrated superior cytotoxic activity compared to etoposide, which served as a standard drug. This reaction is illustrated in Fig. (**15**) [141].

Fathy and colleagues synthesized a range of tetrahydroquinoline derivatives utilizing anisaldehyde (**63**) cyclohexanone (**64**), ethyl cyanoacetate (**65**), an excess of CH_3_COONH_4_ (**66**), and form intermediate 4-(4-methoxyphenyl)-2-oxo-1,2,5,6,7,8-hexahydroq-uinoline-3-carbonitrile **(67**) through a one-pot, four-component reaction. The resulting quinoline derivatives **(68A-D)** demonstrated significant cytotoxic effects across various cell lines, including MCF-7, Hep2, and A549, when compared to 5-FU, which served as a reference drug. This reaction is illustrated in Fig. (**16**) [142]. Abdelmoniem and colleagues synthesized novel hexahydroquinoline derivatives from the sulpha moiety **(69)** and 2-ethylidenemalononitrile **(70)**, utilizing ethanol as a solvent under reflux conditions. The resulting quinoline derivatives, specifically 4-(2-amino-3-cyano-4-aryl-tetrahydroquinolin-1-yl)-N-carbamimidoylbenzenesulfonamides **(71A-D)**, demonstrated the highest cytotoxic activity. This reaction is illustrated in Fig. (**17**) [143]. Several researchers have reported the synthesis of quinoline derivatives using green chemistry approaches and evaluated their anticancer activity (Table **5**).

## 8 COMPARISON BETWEEN CONVENTIONAL METHOD AND GREEN CHEMISTRY APPROACH

Traditional synthetic chemistry methods encompass established practices across multiple disciplines, prioritizing time-honored techniques over contemporary alternatives. In contrast, green (sustainable) chemistry provides a framework for chemical engineers, chemists, and medicinal chemists to develop processes, guidelines, and synthetic methods that support global sustainability efforts. The importance of green chemistry methods in contrast to traditional approaches is outlined below [143-147].

Disadvantages of conventional methods include:

➢ Utilization of toxic chemical substances.
➢ Production of waste materials.
➢ Non-eco-friendly method.
➢ Employment of hazardous materials.
➢ Prolonged reaction times.
➢ High energy consumption.
➢ Advantages of green synthetic approaches include:
➢ Minimal or no toxic chemical reactions using safer, lower-hazard substances, such as water, natural compounds like botanical extracts, and safe separation agents.
➢ Reduction of waste: Most solvents, catalysts, and reagents are recyclable, thereby minimizing unnecessary waste in chemical synthesis.
➢ Utilization of biodegradable materials, such as polymers, that do not persist in the environment, thereby contributes to a cleaner ecosystem.
➢ Mitigation of contamination through ongoing assessment of reaction byproducts and intermediates, when possible, as well as ensuring reactant quality to reduce accidents (fires, explosions, releases).
➢ Application of various selective catalysts, biocatalysts, and nano catalysts that enable higher yields of reaction products in a shorter timeframe.
➢ The green chemistry approach often results in higher-quality products and helps avoid complications with various environmental regulations, making it more cost-effective and efficient.

## CONCLUSION

Cancer continues to be one of the primary causes of mortality globally, highlighting the urgent requirement for more effective treatments utilizing safer molecules. This review presents quinolines as a potential anticancer scaffold, emphasizing its synthetic methods, including conventional or traditional techniques as well as environmentally friendly or green chemistry approaches, along with its anticancer effectiveness. It has diverse structures and the ability to interact with multiple biological targets within cancer cells. Researchers have documented a range of green synthetic methods, such as microwave ultraviolet irradiation-promoted synthesis, green multicomponent one-pot synthesis, and the application of reusable and recyclable green solvents in conjunction with catalysts. Green synthesis techniques offer several benefits compared to traditional methods, including being environmentally friendly, rapid, cost-effective, and requiring fewer hazardous solvents. Furthermore, several disadvantages have been observed, including the lack of suitable alternatives, difficulties in process development, and challenges associated with scaling up laboratory-based processes to industrial production.

The quinoline derivatives synthesized through conventional and green synthetic methods have undergone biological evaluation for their anticancer activity against various cell lines. Common starting materials, such as aniline and isatin, are utilized in the synthesis of the fundamental quinoline ring. Numerous researchers have also examined the SAR of quinoline derivatives. The SAR analysis revealed that substitutions at the 2^nd^, 3^rd^, and 4^th^ positions of the quinoline ring significantly influence anticancer activity. The authors contend that the distinctive properties and anticancer potential of quinolines render them worthy of further exploration, potentially providing new therapeutic avenues for cancer patients and enhancing their prognosis and quality of life in the future.

A major concern for the environment and the methods to prioritize sustainability in public perception is fundamental to the chemical industry, especially in the pharmaceutical and associated industries. This problem has impacted and will continue to shape the methodologies of industrial operations and task execution. Understanding the hazards associated with chemical management and the execution of chemical operations is considered crucial. This also includes an understanding of the concepts of green chemistry. These twelve propositions, formulated over twenty-five years ago, have significantly impacted the comprehension of several chemistry-related matters. They underscore the mitigation of pollution, the minimization of waste generation, and the formulation of intrinsically safe chemical processes. In summary, the concepts underlying these rules are robustly supported by additional principles, collectively aimed at global environmental protection and adherence to sustainable principles in the context of chemistry. This analysis highlights critical topics for future research aimed at improving the use of sustainable materials in pharmaceuticals. These domains include addressing obstacles such as low reaction rates, elevated temperatures, pressures, and energy, as well as advancing more efficient recovery and recycling techniques, which would facilitate the expansion of green chemistry. Future widespread use of green solvents and catalysts is crucial for reducing environmental toxicity.

## STUDY LIMITATIONS

This review thoroughly compiles the existing knowledge regarding both traditional and green synthetic methods for quinoline derivatives and their potential anticancer properties; however, it is important to recognize several limitations. Firstly, a considerable amount of the literature available relies on *in-vitro* studies, with an insufficiency of clinical or *in-vivo* validation, which may not accurately represent the actual therapeutic potential of these compounds within biological systems. Secondly, a publication bias exists that favors the reporting of positive anticancer effects, potentially leading to an overestimation of the true efficacy of certain derivatives. Furthermore, the data gathered from various sources differ in terms of experimental design, the cell lines employed, and the conditions of the assays, which complicates direct comparisons and may introduce inconsistencies in interpretation. From a synthetic perspective, although green chemistry methods provide sustainability advantages, there is a need for further standardization and industrial validation regarding their scalability, reaction efficiency, and product purity. Lastly, more comprehensive SAR studies and mechanistic explorations of certain quinoline series are essential to gain a deeper understanding of the anticancer mechanisms associated with specific quinoline scaffolds. Future research should aim to address these gaps by merging green synthetic advancements with thorough preclinical and clinical assessments to translate laboratory discoveries into practical therapeutic agents effectively.

## Figures and Tables

**Fig. (1):**
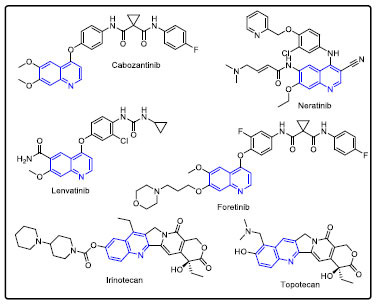
The chemical structures of quinoline based marketed drug.

**Fig. (2):**
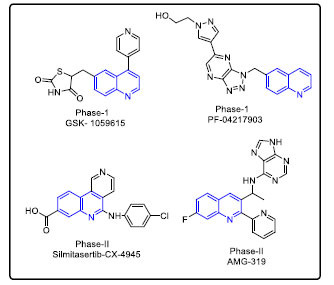
The structures of quinoline based drug in clinical trial.

**Fig. (3):**
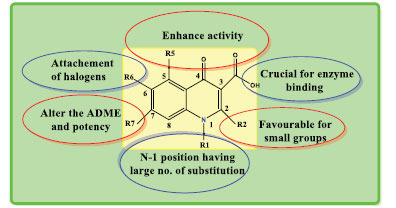
General SAR of quinoline moiety.

**Fig. (4):**
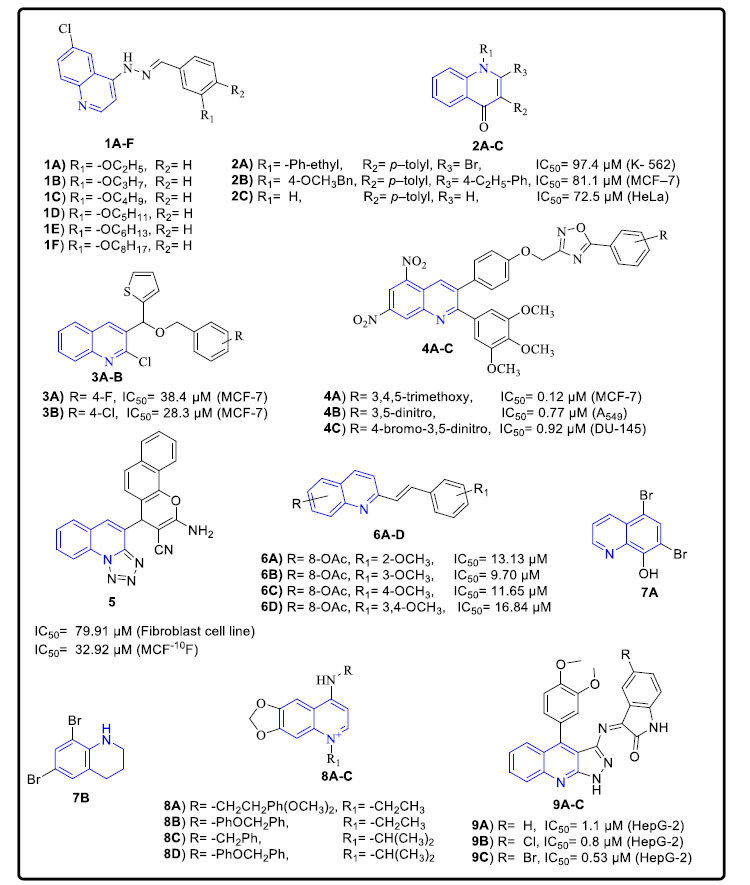
Structures of quinoline and its derivatives **(1-9)** showing anticancer activity.

**Fig. (5):**
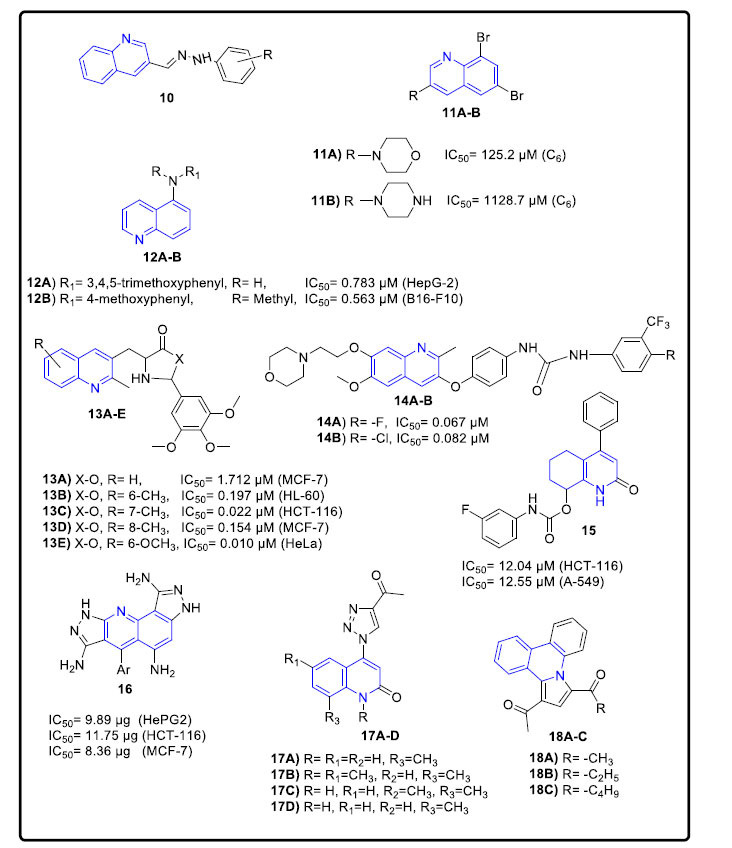
Structures of quinoline and its derivatives **(10-18)** showing anticancer activity.

**Fig. (6):**
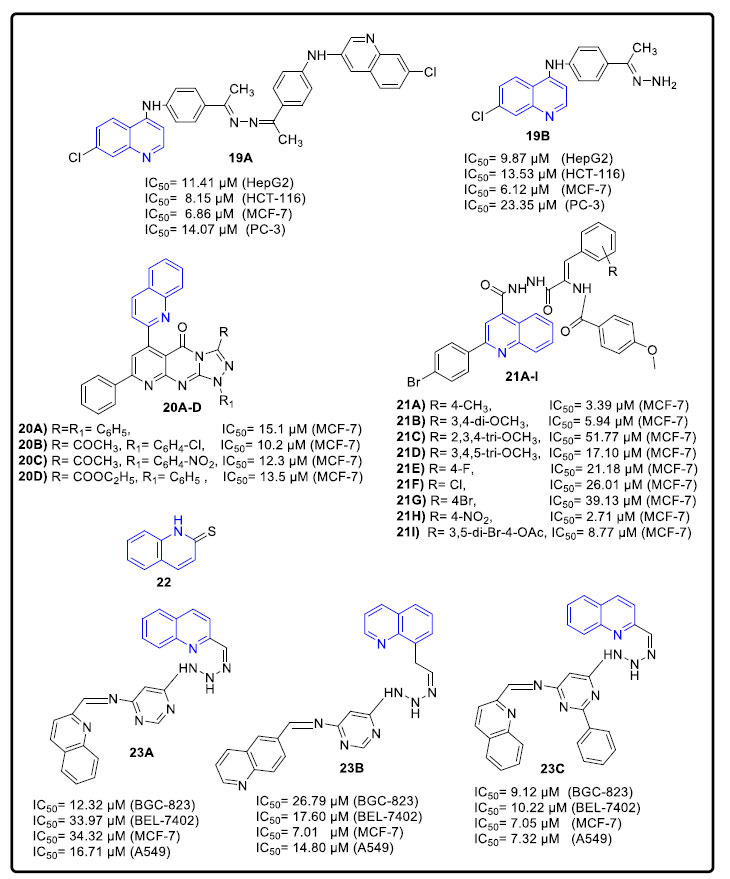
Structures of quinoline and its derivatives **(19-23)** showing anticancer activity.

**Fig. (7):**
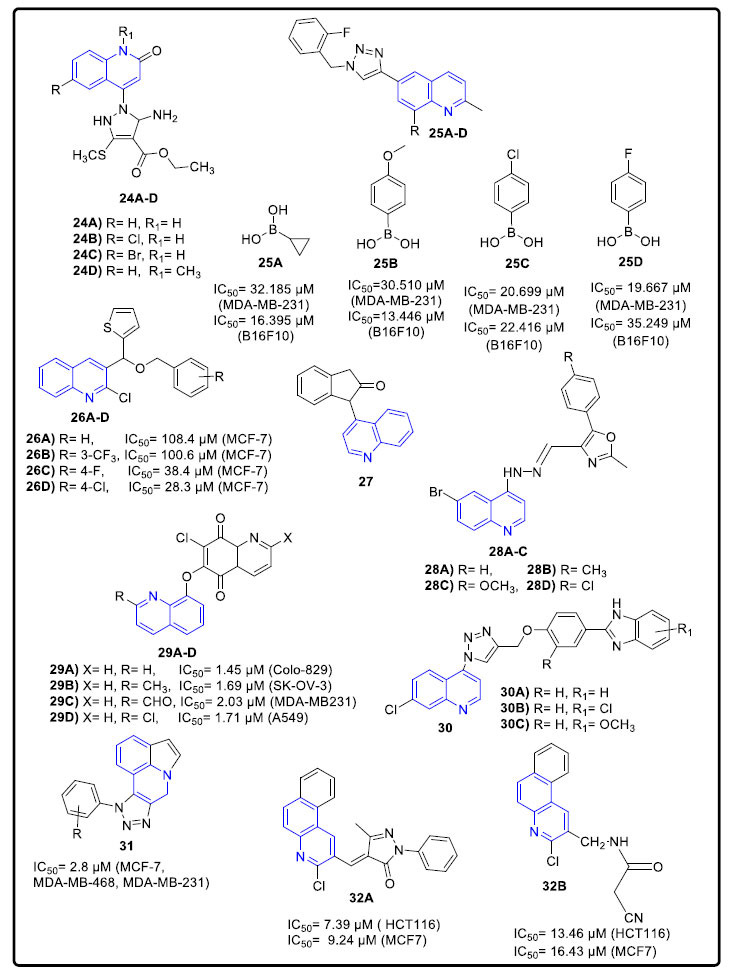
Structures of quinoline and its derivatives **(24-32B)** showing anticancer activity.

**Fig. (8):**
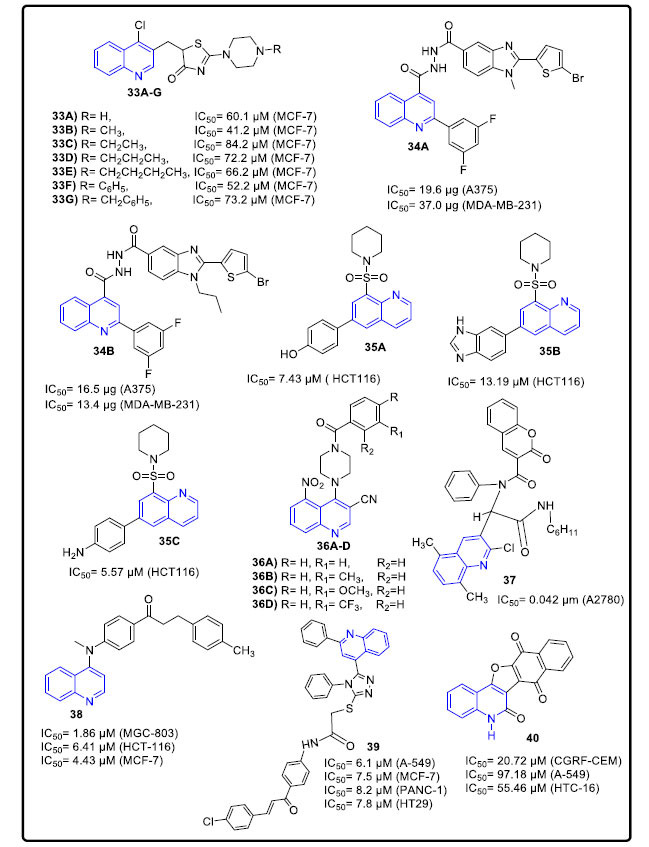
Structures of quinoline and its derivatives **(33-40)** showing anticancer activity.

**Fig. (9):**
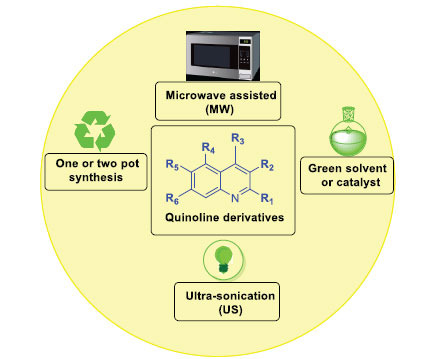
Various green synthesis approaches for quinoline derivatives as anti-cancer agents.

**Fig. (10):**
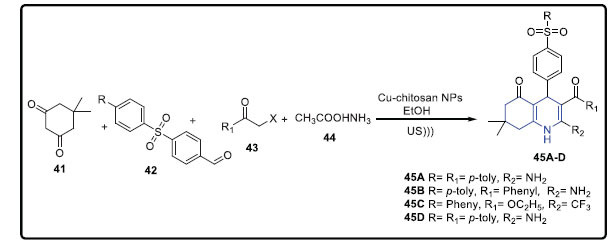
Synthesis of 4,6,7,8-tetrahydroquinolin-5(1H)-one derivatives.

**Fig. (11):**
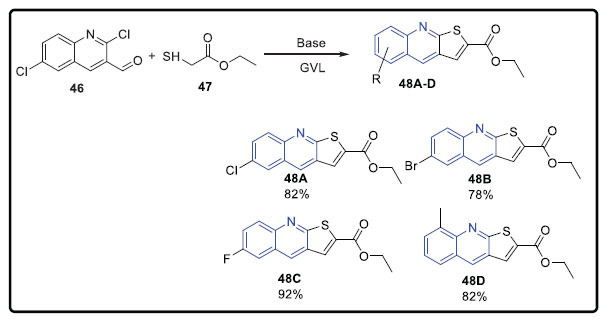
Synthesis of quinoline derivatives.

**Fig. (12):**
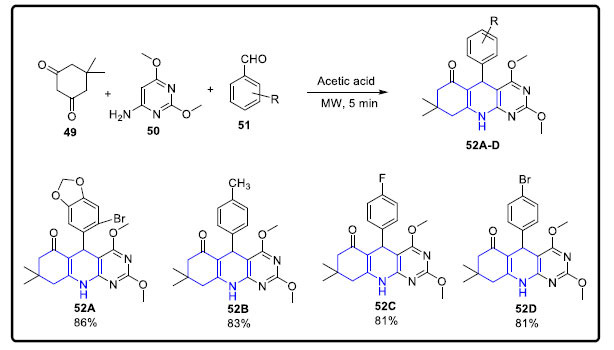
Synthesis of 5,8,9,10-tetrahydropyrimido[4,5-b]quinolin-6(7H)-one derivatives.

**Fig. (13):**
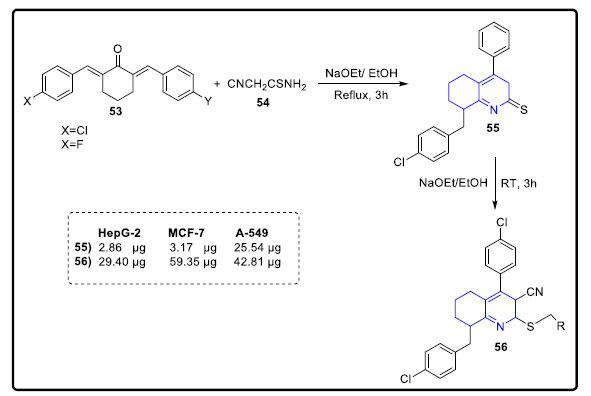
Synthesis of tetrahydroquinoline quinoline derivatives.

**Fig. (14):**
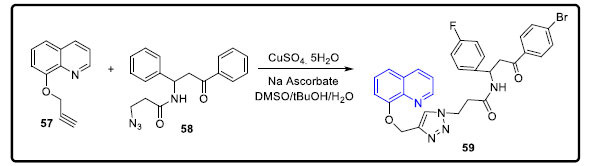
Synthesis of quinoline-triazolehybrid.

**Fig. (15):**
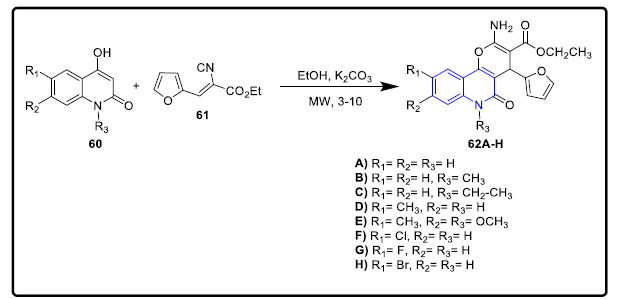
Synthesis of pyrano[3,2-c]quinoline-3 carboxylates derivatives.

**Fig. (16):**
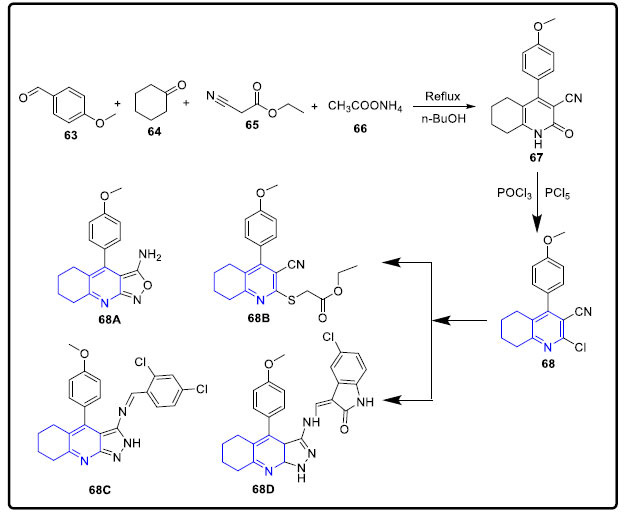
Synthesis of tetrahydroquinoline derivatives.

**Fig. (17):**
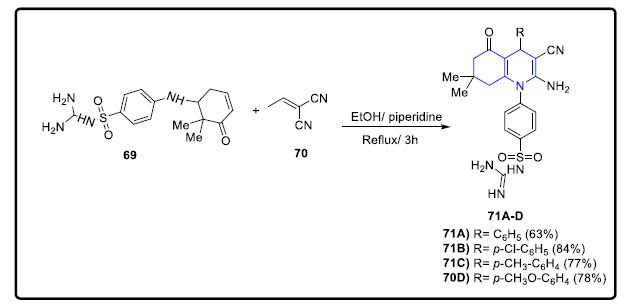
Synthesis of quinoline derivatives.

**Table 1:** Physical and chemical properties of quinoline.

**Parameter**	**Physical Properties**	**Parameter**	**Chemical Properties**
IUPAC name	Benzo[b]pyridine	Nature	Basic character
Chemical formula	C_9_H_7_N	Reaction	➢ Electrophilic substitution reaction (at C-5 and C-8)
Molar mass	129.16 g/mol		➢ Nucleophilic substitution’s reaction (C-2 or at C-4 if C-2 is blocked)
Appearance	Colorless and oily liquid		➢ Oxidation and reduction
Density	1.093 g/mL		➢ Reaction with alkyl halides
Melting point	-15°C	Structure	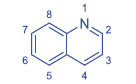
Boiling point	237°C		
Solubility	Slightly soluble in water and soluble in ether, alcohol, and carbon disulfide		

**Table 2:** SAR of quinoline derivatives as anticancer agents.

**S. No.**	**Structure**	**SAR**	**Reference**
1.	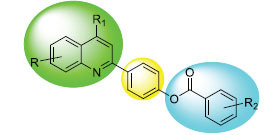	**Quinoline ring**- Incorporating a chloro group at an appropriate location on the quinoline ring increases its anticancer efficacy. **Phenyl ring-** modifications at the para position significantly boost its activity. **Substituting various groups, such as CH_3_, Cl, and CF_3_, on the benzene ring with a benzoate group** is crucial for enhancing anticancer properties.	[55]
2.	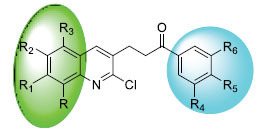	**Quinoline ring** - It is advisable to attach the methoxy or phenoxy group at appropriate positions, such as R_1_, R_2_, R_3_, or R_4_, to improve cytotoxic efficacy. **Phenyl ring-** The introduction of the methoxy group at the specified position has demonstrated significant anticancer activity.	[56]
3.	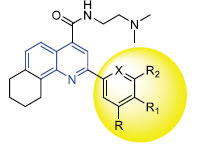	● Substitution of the 2^nd^ position at the quinoline ring increases the anticancer activity. ● Substitution of the electron-donating group increases the cytotoxic activity.	[57]
4.	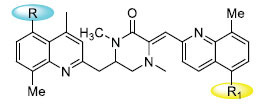	**R and R_1_-**Replacing H with NO_2_ has been demonstrated to be a more effective anticancer agent.	[58]
5.	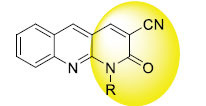	The presence of the CN group and the modification of the R-position enhance the anticancer efficacy.	[59]

**Table 3:** Traditional methods and name reaction for the synthesis of quinoline and its derivatives.

**Name** **Reaction**	**Substrate**	**Reactant**	**Catalysts**	**Product**
Skraup synthesis	Aniline	Glycerol	H_2_SO_4_, PhNO_2_	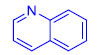
	Glycerol and aniline are combined and heated in the presence of sulfuric acid along with a mild oxidizing agent, usually arsenic pentoxide or nitrobenzene. This reaction is exothermic and has the potential to become extremely violent; therefore, substances such as boric acid or ferrous sulfate are commonly employed to mitigate the effects. 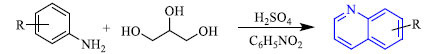			
Doebner Miller reaction	Aniline	α, β-unsaturated carbonyl compound	*p*-TSA	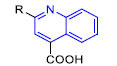
	Quinoline derivatives are formed through the reaction of an aniline with α, β-unsaturated carbonyl compounds, facilitated by *p*-toluene sulfonic acid (*p*-TSA) or a proton oxidant. 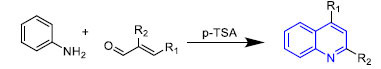			
Doebner synthesis	Aniline	Pyruvic acid and aldehyde		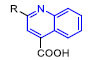
	Quinoline-4-carboxylic acid is produced through a three-component coupling reaction involving an aniline, an aldehyde, and pyruvic acid. 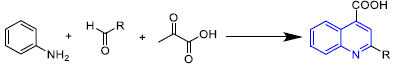			
Riehm Synthesis	Aryl amine	Ketones	AlCl_3_	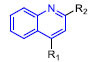
	Extended heating of arylamines and ketones, with or without the inclusion of aluminum chloride or phosphorus pentachloride, leads to the formation of quinoline derivatives. 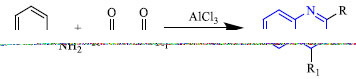			
Gould Jacob synthesis	Aniline	Ethyl ethoxymethylenemalonate	R-OH	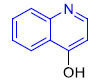
	Aniline is condensed with ethyl ethoxymethylenemalonate. For anilines having electron-donating groups at the *m*-position, it works well. 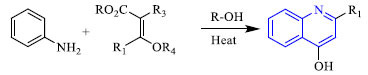			
Conrad Limpach synthesis	Primary aromatic amine	β-ketoester	H_2_SO_4_	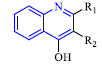
	The synthesis of 4-hydroxyquinolines through a Schiff base involves the combination of addition and rearrangement condensation reactions between anilines and β-ketoesters. 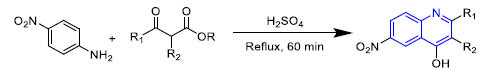			
Combes’s synthesis	Aniline	β-diketone	H_2_SO_4_	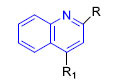
	In this process, a diketone, dialdehyde, or aldehyde reacts with an arylamine (specifically aniline) to form an enamine. Upon heating the reaction mixture with H_2_SO_4_, cyclodehydration occurs, resulting in the formation of quinoline derivatives. 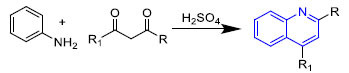			
Pfitzinger reaction	Isatin	Carbonyl compound	KOH	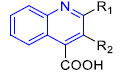
	When a base, like potassium hydroxide, is present, isatin undergoes a chemical reaction with a carbonyl compound, resulting in the formation of substituted quinoline-4-carboxylic acids. 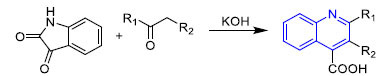			
Friedlander synthesis	2-Aminobenzaldehyde	Acetaldehyde	NaOH	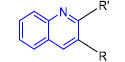
	In this reaction, *o*-amino benzaldehyde undergoes condensation with acetaldehyde in the presence of sodium hydroxide. 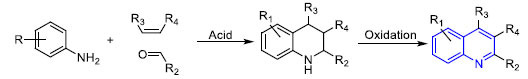			
Povarov reaction	Aromatic imine	Alkene (etho/ether/enamine)	Lewis’s acids, Boron trifluoride	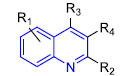
	The reaction between aniline and benzaldehyde produces a Schiff base, which is subsequently followed by a cycloaddition involving an aromatic imine and an alkene. 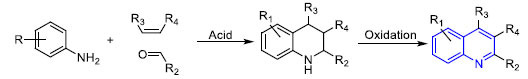			
Knorr synthesis	β-ketoanilide	-	H_2_SO_4_,	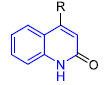
	This process involves the transformation of a β-ketoanilide into a 2-hydroxyquinoline using H_2_SO_4_. 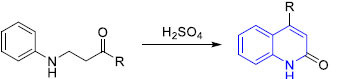			
Camps synthesis	*o*-acylamino acetophenone	-	NaOH	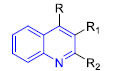
	In this reaction, hydroxide reacts with o-acylamino acetophenone to yield quinoline. 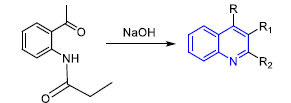			
Niemento-wski synthesis	Anthranilic acid	Carbonyl compound	-	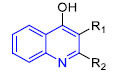
	A quinoline compound is produced when anthranilic acid interacts with a carbonyl compound. 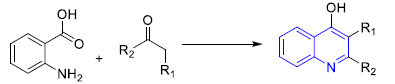			

**Table 4:** Various green synthesis Techniques with advantages and drawbacks.

**Technique/Approach**	**Advantages**	**Drawback**	**References**
Microwave-Assisted Synthesis	● High yields ● Short reaction time ● Energy efficient	● Local overheating possible ● Need specific equipment and apparatus	[130]
Ultrasound-Assisted Synthesis	● Energy efficient ● Accelerates reaction rate ● Minimized accidental cases ● Easy to handle	● Not scalable	[131]
One or Two pot synthesis	● Atom economy ● Prevention of by -products or waste products	● Need to perform specific optimization of the reaction	[132]
Green solvent or catalyst	● Low or non-toxic ● Cast saving ● Good yields	● Limited substrate scope	[133]
Biocatalysis	● Highly selective ● Low environmental impact	● Stability issues ● High cost of enzymes	[134]
Solvent or catalyst-free	● Reducing the use of auxiliary materials	● Limited conversion rates	[135]

**Table 5:** Summary of green chemistry approaches for anticancer quinoline derivatives.

**Authors**	**Technique/ Approach**	**Solvent**	**Catalyst**	**Reaction Time**	**Yield (%)**	**References**
Mokhtar *et al*., 2021	Ultrasonic irradiation	Ethanol	CS/Cu-NPs	20-30 min	90-95	[136]
Mu *et al*., 2021	Green solvent	gamma-valerolactone	NA	1h	78-92	[137]
Patel *et al*., 2022	Microwave assisted	NA	Glacial acetic acid	5 min	81-86	[138]
Alanazi *et al*., 2022	Ultrasonic irradiation	Ethanol/ H_2_O	NaOH/Ionic liquids	5-60 min	87-95	[139]
Ramya *et al*., 2024	Stirrer	DMSO/ H_2_O	Copper sulphate/ tBuOH	12h	87	[140]
Aly *et al*., 2025	Microwave assisted	Ethanol	potassium carbonate	3-10 min	78-90	[141]
Fathy *et al*., 2021	One-pot reaction	Solvent free	POCl_3_/PCl_5_	-	-	[142]
Abdelmoniem *et al*., 2025	Reflux	Ethanol	Piperidine	3h	63-84	[143]

## LIST OF ABBREVIATIONS

SAR: Structure-activity relationship
QSAR: Quantitative structure-activity relationship
µg: Microgram
µM: Micromolar
Eq: Equivalent
MIC: Minimum inhibitory concentration
IC_50_: Half-maximal inhibitory concentration
MTT: 3(4,5- dimethythiazol-2-yl)-2,5-diphenyl tetrazolium bromide
MW: Microwave-assisted
US: Ultrasound-assisted
